# Medical student, nursing student, and non-health care respondents' implicit attitudes toward doctors and patients: Development and a pilot study of a new implicit attitudes test (IAT)

**DOI:** 10.1371/journal.pone.0183352

**Published:** 2017-08-15

**Authors:** Alan Schwartz, Abdelhamid Mazouni

**Affiliations:** Department of Medical Education, University of Illinois at Chicago, Chicago, Illinois, United States of America; La Trobe University, AUSTRALIA

## Abstract

**Introduction:**

Medical educators have been concerned that medical students may decline in empathy for patients during the course of their training, based on studies measuring clinical empathy using psychometrically strong self-report measures. Clinical empathy is a complex construct, incorporating attitudes toward patients but also other components, such as professional detachment. Triangulation of extant measures with instruments based on nonreactive methods could provide a better understanding of whether and how physician attitudes toward patients may be changing during training. We sought to develop and pilot-test such a nonreactive method.

**Methods:**

We develop variations of an implicit association test (IAT) designed to measure attitudes toward physicians and patients based on speed of reaction to images of actors and positive and negative words. In the IATs, the same actors are photographed as doctors, clinic outpatients, hospitalized inpatients, and as a “general public” control. We examine preliminary evidence for their validity by collecting pilot data from internet participants (not involved in the health professions), medical students, and nursing students.

**Results:**

Internet participants (n = 314) and nursing students (n = 31) had more negative associations (IAT scores) with doctors than did medical students (n = 89); nursing students and female internet participants had more positive associations with hospitalized patients than did medical students and male internet participants. Medical students’ associations with hospitalized patients varied by year of training.

**Discussion:**

This IAT may provide insight into implicit attitudes among those who enter training for the health profession and changes in those attitudes that may be inculcated during that training.

## Introduction

In the past decade, medical educators have been concerned that medical students may decline in empathy for patients during the course of their training [1, 2]. A common element in nearly all of these studies is the use of the Jefferson Scale of Physician Empathy–Student version (JSPE-S), a self-report survey instrument with good psychometric properties [3–6]. JSPE-S measures (as well as other self-report empathy measures, such as the Interpersonal Reactivity Inventory [7]) have been found to differ by gender (women scoring higher for empathy) and medical specialty (trainees in “people-oriented” specialties scoring higher than those in “technology-oriented” specialties) in several countries. [3, 4, 8–12] On the other hand, JSPE-S scores in medical students have been reported to decline over time in training in some studies and to increase in others. [1, 10, 13, 14]; although first-year nursing students reportedly score higher on empathy than first-year medical students, similarly conflicting evidence has been reported in nursing students. [15–17] JSPE-S’s construct, clinical empathy, involves both traditional empathic components (such as positive attitude and perspective-taking) and recognition of the need for professional detachment and the portrayal of positive attitudes, regardless of the physician’s actual feeling [18]. Triangulation of extant measures with instruments based on nonreactive methods could provide a better understanding of whether and how physician attitudes toward patients may be changing during training.

The Implicit Association Test (IAT) [19] is a measure of attitudes based on reaction times to a categorization task. Typically, IATs measure the association between category members and words reflecting positive and negative feelings by asking participants to classify pairs consisting of a category member or non-member and a positive or negative feeling word into category member or feeling categories, and tracking the speed at which they do so. Faster classification of member/negative and non-member/positive pairs than of member/positive and non-member/negative pairs suggest that members are more easily associated with more negative attitudes than non-members. For example, an IAT may demonstrate that respondents are faster (slower) to associate images of European-Americans with positive (negative) words than they are to associate images of African-Americans with positive (negative) words, and explain this latency difference by additional cognitive processing required to overcome a latent association between African-Americans and negative concepts. IATs have been useful in identifying attitudes that are socially sensitive (e.g. racial bias) or about which participants may not be explicitly aware, and have also been used as educational interventions by inviting participants to reflect on their results [20, 21].

As the primary purpose of this exploratory study, we developed an innovative set of IATs for measuring positive or negative attitudes toward patients as a category of persons. We report on the development of the measure and initial results from its administration to a group of internet users not involved in health care, medical students, and nursing students. In describing the results, we compare responses by respondent gender, respondent white/non-white race, and, for students, by year in training. A secondary purpose was to explore the initial results from the standpoint of two initial hypotheses: we expected to see, on average, more implicitly negative associations (IAT scores) with doctors relative to patients or the public among non-health-care students, and more implicitly negative associations with patients relative to doctors or the public among medical students. We report all measures, manipulations, and exclusions in these studies.

## Materials and methods

### Development of the IATs

As with other IATs, we paired words with positive and negative valence with images of members of two different categories (e.g. doctors and patients).

#### Word stimuli

We selected positive/negative words from an available database of emotional evaluations of nearly 14,000 English words [22]. We chose words of extreme but approximately inverse valence and similar arousal levels between the positive and negative words, excluding words directly related to illness or medical care. Table 1 displays the selected words.

**Table 1:** Positive and negative words used to construct IAT stimuli.

Word	Valence (-4 to +4)	Arousal (1–7)
Fun	3.37	6.32
lovable	3.26	5.41
joy	3.21	5.55
relaxing	3.19	4.29
sunshine	3.14	5.32
excited	3.11	6.43
wreck	-3.38	4.65
disaster	-3.29	6.35
nightmare	-3.21	5.83
stress	-3.21	4.72
unhappy	-3.16	5.10
worthless	-3.11	4.45

#### Image stimuli

We photographed six actors (three men and three women, with one White, African-American, and Hispanic within each gender). We sought to control for attitudes toward individual actors or actor characteristics (e.g. race or gender) by using the same actors in the images in each category, with differences in dress and setting conveying category membership. Each actor was photographed in four poses: as an inpatient (in a hospital bed, wearing a gown, with an IV stand and other medical equipment nearby), as an outpatient (sitting on an exam room table, wearing a gown, with a blood pressure gauge and otoscope on the wall), as a physician (wearing a white coat, with a stethoscope around the neck, carrying a clipboard), and as a neutral "member of the public" (standing indoors next to a potted plant and a painting). In all poses, actors were instructed to use a neutral facial expression. Photos were cropped and resized to 300x200 pixels for on-screen display.

#### IAT design

Four different IATs were designed by combining the common word stimuli with four different pairings of image stimuli: inpatient vs. doctor, outpatient vs. doctor, public vs. doctor, and inpatient vs. public.

As with previously developed IATs, each of our IATs consisted of 7 task blocks. Table 2 illustrates the structure of the task blocks. In Block 1, images of one category (e.g. inpatients) were assigned to the left-key response, while images of the other (e.g. doctors) were assigned to the right-key response. For Block 2, positive words were assigned to the left-key response and negative words were assigned to the right-key response. Blocks 3 (practice) and 4 (test) each consisted of positive words or images of the first category being assigned to the left-key response, and negative words or images of the second category being assigned to the right-key response. In Block 5, the items assigned to the response keys were opposite of those in Block 1; that is, images of the second category were assigned to the left-key response, whereas images of the first category were assigned to the right-key response. In Block 6 (practice) and 7 (test), the items assigned to the left and right key responses were opposite to those in Blocks 3 and 4; that is, positive words or images of the second category were assigned to the left-key response, and negative words or images of the first category were assigned to the right-key response. Blocks 1, 2, and 5 consisted of 20 trials; blocks 3, 4, 6, and 7 consisted of 40 trials.

**Table 2:** Example of IAT task block structure.

Block	Stimuli	Press left key for…	Press right key for…	Trials
1	Images of inpatients and doctors	Inpatient	Doctor	20
2	Words	Positive word	Negative word	20
3	Images and words	Inpatient or positive word	Doctor or negative word	40
4	Images and words	Inpatient or positive word	Doctor or negative word	40
5	Images of inpatients and doctors	Doctor	Inpatient	20
6	Images and words	Doctor or positive word	Inpatient or negative word	40
7	Images and words	Doctor or positive word	Inpatient or negative word	40

#### IAT software

**Fig 1:**
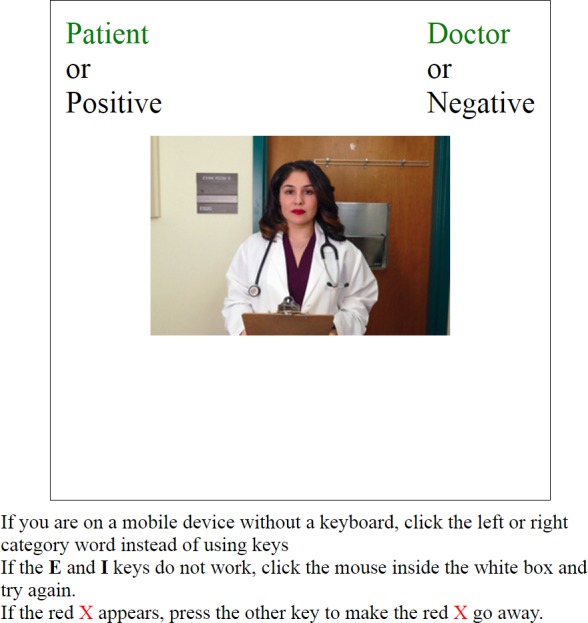
Screenshot of a trial. This example trial is from the outpatient vs. doctor condition, block 3, Hispanic woman doctor. The individual pictured has given written informed consent (as outlined in PLOS consent form) to publish this image.

We implemented the IATs using WebIAT, open source web-based IAT software. [23], modified to allow for the IAT to be completed on a mobile device with a touch screen rather than requiring a keyboard. WebIAT requires a correct response at each trial to proceed to the next. Accordingly, errors result in slower trial latencies, and we incorporated this in the scoring of the IATs as described below. Fig 1 show an example of an IAT trial in block 3 of the outpatient vs. doctor condition.

### Participants and setting

We recruited three groups of participants: Amazon Mechanical Turk (AMT) workers not involved in health care, medical students, and nursing students. In recruitment materials, the study was described as one asking participants to “classify images into categories”. As we were developing a new measure, did not have estimates of effect size, and did not plan to conduct substantive statistical tests, samples sizes were based on general recommendations for IAT sample sizes [24] and availability of resources to compensate subjects rather than power calculation.

We screened 700 AMT workers with a short questionnaire asking for gender, age, ethnicity, and field of work or study. Participants could select multiple ethnicities and multiple fields of work or study. We excluded workers who indicated that their field of work or study related to "health care or social assistance". The remaining workers were randomly assigned to one of the four IATs and then sampled to invite them to complete their assigned IAT. We invited all workers who did not report White ethnicity and a random subset of workers who reported White ethnicity, continuing invitations until we reached at least 80 workers per IAT (320 participants total). Workers were paid $0.05 for participating in the screening questionnaire, and $5 for completion of an IAT. Data collection ran from June 20, 2016 to June 28, 2016.

We sought to recruit 80 medical students (completing a 4-year Medical Doctor (MD) program) and 40 nursing students (completing a 2-year Bachelor of Science in Nursing (BSN) program) from the University of Illinois at Chicago Colleges of Medicine and Nursing, stratified by year in school (1–4 for medical students, 1–2 for nursing students) and randomly allocated within stratum between two of the IAT conditions (outpatient vs. doctor and inpatient vs. public) using a proportional randomization algorithm (in which probability of allocation to each condition was proportional to the number of unfilled slots for that condition), to obtain 10 medical students and 10 nursing students per year per IAT. We limited the student study to two conditions and 120 students due to resource constraints. To maintain anonymity, no demographics other than year in school were collected from students. Students were contacted by flyers posted around the schools and distributed to student electronic mailing lists. Students were paid $15 for completion of an IAT. Data collection took place September-November 2016.

The UIC IRB determined that this study was exempt from review and did not require written consent. Participants were provided with on-screen informed consent information and were not required to document consent other than by continuing with the study.

### IAT scoring

We scored the IAT using the improved algorithm recommended by Greenwald, Nosek, and Banaji [24]. Specifically, we combined data from both practice blocks (blocks 3 and 6) and test blocks (blocks 4 and 7), eliminated trials with latencies > 10,000ms, eliminated subjects with more than 10% of trials with latencies < 300ms (suggesting they were simply pushing keys at random as quickly as possible), and computed the recommended D score as the difference in latencies between the two practice blocks and the difference in latencies between the two test blocks divided by the pooled standard deviations for the trials in each pair of blocks. This algorithm is suggested for IATs like ours in which participants are forced to correct errors.

In our IATs that include doctors, positive D values reflect implicitly more negative associations with doctors (relative to inpatients, outpatients, and the public, respectively). In the IAT including images of the general public and inpatients, positive D values reflect implicitly more positive associations with inpatients (relative to the general public).

### Data analysis

We were most interested in describing observed effect sizes for future studies rather than in testing hypotheses about differences, but we conducted a small number of hypothesis tests. We expected to see, on average, more implicitly negative associations with doctors relative to patients or the public among AMT workers, and more implicitly negative associations with patients relative to doctors or the public among medical students. Because we did not have any a priori reason to assume a normal or other parametric distribution of IAT D-scores, we compared the median IAT D-scores to the value 0 using one-sample non-parametric Wilcoxon signed-rank tests. We compared IAT D-scores among AMT respondent demographic groups (by gender and by white/no-white), among respondent samples (AMT workers, medical students, nursing students), and among years of medical school students (1–4) and nursing school students (1–2) using Wilcoxon rank-sum tests. Comparisons between nursing students and medical students (the smallest samples) had 83% power to detect a D-score difference of 0.20; comparisons by gender or race among AMT workers had over 95% power to detect a similar difference. Statistical tests were performed using R 3.2. [25].

## Results

### Participants

We recruited and randomized 320 Amazon Mechanical Turk workers. Six subjects were excluded due to >10% of trials with latencies < 300ms. Among the remaining subjects, 7 trials were dropped due to very long (>10,000ms) latencies.

For medical and nursing students, experiment slots were considered unfilled until an IAT was completed, and multiple students could begin an IAT at the same time. As a result, we collected data from 89 medical students and 31 nursing students. One additional nursing student was excluded due to >10% of trials with <300ms latency.

Table 3 shows the demographics of the respondents in each condition.

**Table 3:** Demographics of participants. MD1-MD4 refers to the four years of medical school toward obtaining the medical doctor degree. BSN1-BSN2 refers to the two years of nursing school toward obtaining a bachelor of science in nursing degree.

	Overall	Condition 1 (Inpatient vs. Doctor)	Condition 2 (Outpatient vs. Doctor)	Condition 3 (Public vs. Doctor)	Condition 4 (Inpatient vs. Public)	p
AMT workers						
N	314	79	78	78	79	
Women (%)	153 (48.7)	36 (45.6)	37 (47.4)	40 (51.3)	40 (50.6)	0.88
Age (mean years, (sd))	35.0 (11.5)	33.0 (11.1)	36.3 (11.5)	34.7 (12.3)	36.0 (11.1)	0.26
Race (%)						0.29
African-American	23 (7.3)	5 (6.3)	9 (11.5)	4 (5.1)	5 (6.3)	
American Indian	1 (0.3)	1 (1.3)	0 (0.0)	0 (0.0)	0 (0.0)	
Asian	34 (10.8)	12 (15.2)	5 (6.4)	11 (14.1)	6 (7.6)	
Decline to state	1 (0.3)	0 (0.0)	0 (0.0)	1 (1.3)	0 (0.0)	
Hawaiian	2 (0.6)	0 (0.0)	0 (0.0)	0 (0.0)	2 (2.5)	
Hispanic	19 (6.1)	4 (5.1)	8 (10.3)	3 (3.8)	4 (5.1)	
Multi	14 (4.5)	5 (6.3)	3 (3.8)	4 (5.1)	2 (2.5)	
White	220 (70.1)	52 (65.8)	53 (67.9)	55 (70.5)	60 (75.9)	
Medical Students						
n	89		44		45	
Medical school year						
MD1	20		10		10	
MD2	21		11		10	
MD3	23		11		12	
MD4	25		12		13	
Nursing Students						
n	31		15		16	
Nursing school year						
BSN1	20		10		10	
BSN2	11		5		6	

### IAT scores

Error rates in classifying images were small. Across all trials in block 1 (classify image alone) in all conditions, errors were made on 2.8% of trials; for comparison, across all trials in block 2 (classify words alone) in all conditions, errors were made on 4.3% of trials. The highest rate of error was observed in condition 4 (inpatient vs. public), where errors were made on 3.8% of image-alone trials and 4% of word-alone trials. A logistic mixed effects model fitted to trials in conditions 2 and 4, with fixed effects of participant group (AMT vs. medical student vs. nursing student), block (1 vs 2), condition (2 vs. 4), and the block x condition interaction, and a random effect of participant, confirmed that errors were equally likely for words and images in condition 4 (inpatient vs. public) but less likely for images than words in condition 2 (doctor vs. outpatient), with no effect of participant group.

Table 4 shows the distribution of mean and median IAT scores by respondent group and condition.

**Table 4:** Distribution of IAT scores by respondent group and condition.

	Condition 1 (Inpatient vs. Doctor)		Condition 2 (Outpatient vs. Doctor)		Condition 3 (Public vs. Doctor)		Condition 4 (Inpatient vs. Public)	
	Mean (sd)	Median	Mean (sd)	Median	Mean (sd)	Median	Mean (sd)	Median
AMT workers	0.03 (0.33)	0.02	0.11 (0.35)	0.22	0.17 (0.37)	0.17	0.07 (0.41)	0.03
Medical students			-0.01 (0.38)	-0.04			0.12 (0.39)	0.03
Nursing students			0.19 (0.31)	0.24			0.30 (0.37)	0.31

Overall, 23 respondents (AMT workers, 9 medical students, 6 nursing students) completed the IAT in any condition using a mobile device, and these respondents had longer and more variable latencies overall (across blocks 4 and 7, average latency for mobile respondents was 1389ms (sd = 394), and average latency for desktop respondents was 919ms (sd = 211), Wilcoxon rank-sum W = 1212, p < .001). However, because IAT scores are standardized within respondent and each respondent uses a single device during the IAT, overall differences in latency between devices should not affect IAT scores, and indeed, IAT scores across all conditions did not vary by whether the IAT was performed on a mobile device or not (Wilcoxon rank-sum W = 3960, p = .19). The same pattern was found when limiting the analysis to only Conditions 2 and 4.

#### AMT workers

Median AMT workers’ IAT D-scores were significantly different from zero in two conditions. In the outpatient vs. doctor condition (condition 2), AMT workers more quickly associated the doctor with negative words (and the patient with positive) than the reverse (D = 0.22, W = 2143, p = .003). The same effect was found in the public vs. doctor condition (condition 3) (D = 0.17, W = 2272, p = .003). AMT workers overall did not have different implicit associations for inpatients vs. doctors (p = .46) or inpatients vs. public (p = .18).

These effects did not differ by the gender or race (white or non-white) of the respondents, with one exception. In the inpatient vs. public condition (condition 4), male respondents did not show significantly different implicit associations (median D = 0.01), but female respondents were significantly faster at associating inpatients with positive words (and the public with negative) than the reverse (median D = 0.15; Wilcoxon rank-sum test between male and female respondents W = 541, p = .02).

#### Medical and nursing students

**Fig 2:**
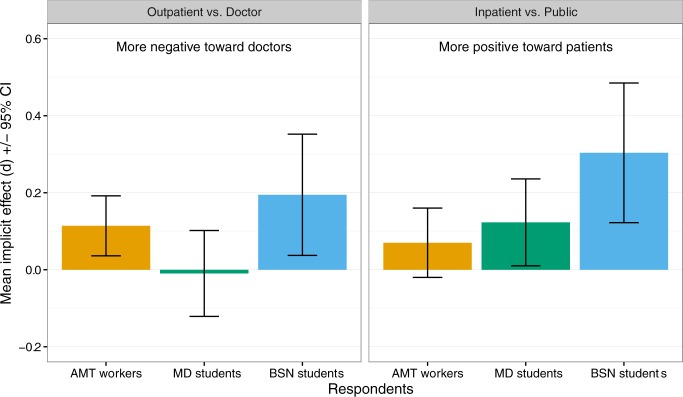
Comparison of attitudes by respondent group for the outpatient vs. doctor and inpatient vs. public study conditions. Bars are mean implicit effects; error bars show 95% confidence intervals.

Overall, both nursing students and AMT workers had more negative implicit associations with physicians (vs. outpatients) than did medical students in condition 2 (confirming the first of our tentative hypotheses), while nursing students had more positive implicit associations with inpatients (vs. public) than did medical students or AMT workers in condition 4 (disconfirming our second hypothesis that medical students would have less positive implicit associations with inpatients than AMT workers; see Fig 2).

**Fig 3:**
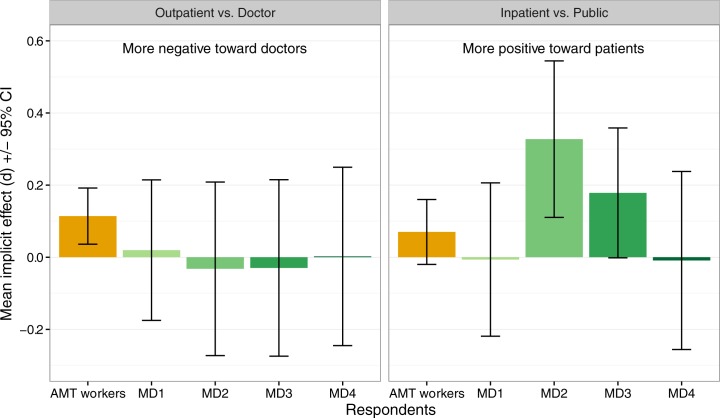
Comparison of medical student attitudes by student year for the outpatient vs. doctor and inpatient vs. public study conditions. Bars are mean implicit effects; error bars show 95% confidence intervals.

Despite a lack of power due to small sample sizes, differences were also observed within medical students by year of training (Fig 3). Medical students did not differ by year of training in condition 2 (outpatient vs. doctor), but had stronger implicit positive associations with inpatients (vs. public) at the start of their second year than at the start of their first year (p = .03) or fourth year (p = .04).

**Fig 4:**
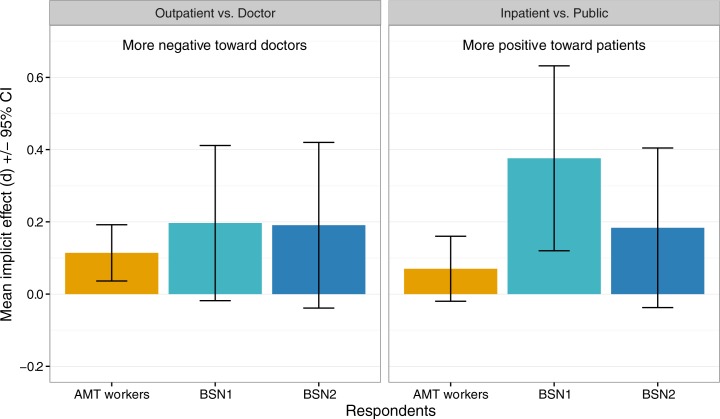
Comparison of nursing student attitudes by student year for the outpatient vs. doctor and inpatient vs. public study conditions. Bars are mean implicit effects; error bars show 95% confidence intervals.

A similar examination of nursing students by year found that students at the start of their first year did not have significantly stronger implicit positive associations with inpatients (vs. public) than students in their second year (Fig 4), despite a similar pattern (this comparison was likely underpowered).

## Discussion

We developed a set of new IATs designed to measure implicit attitudes toward patients and physicians, and collected pilot data about these attitudes among internet participants not involved in health care, medical students, and nursing students.

Internet participants had negative associations with doctors relative to non-doctors. These negative attitudes may echo explicit survey findings that Americans, though generally satisfied with the care they receive from their own doctor, express low overall public trust in doctors as a group. [26] Attitudes toward hospitalized patients were more complex; these patients appeared to be regarded more positively than doctors only by female respondents. At the same time, for both male and female respondents, hospitalized patients were regarded no more or less positively than members of the public when included together in an IAT. This influence of the comparator or choice set included in the IAT suggests that further examination of differences in implicit attitude by respondent gender may be warranted, as this represents a substantial point of departure from self-reported empathy measures, which nearly always find higher empathy scores in women than men.

We administered two of the IATs to medical and nursing students, stratified by year in school. Patterns in the findings lend credence to the meaningfulness and utility of the instruments. Differences by group were sensitive to the IAT condition and sensible: medical students had the least negative associations with doctors (as hypothesized) and nursing students had the most positive associations with hospitalized patients. Nursing is an inherently “people-oriented” profession, while medical students may intend to enter into either “people-oriented” or “technology-oriented” specialties, and explicit measures of clinical empathy has been found to be higher in nursing students than medical students and higher in “people-oriented” than “technology-oriented” medical specialties. [4, 15] Contrary to our hypothesis, medical students did not display less positive associations with hospitalized patients than AMT workers did. Differences in attitudes toward patients based on training year in medical and nursing students were also suggestive changing attitudes toward patients in different years of training, which may contribute to (or reflect) reported changes in empathy in these students, particularly as they enter their clinical years. [14, 15] This IAT may provide insight into implicit attitudes among those who enter training for the health profession and changes in those attitudes during that training.

Because the focus of this pilot study was instrument development and initial testing, resources were limited and the student samples are small, self-selected, and from a single U.S. University. The non-health-care student sample was a convenience sample recruited from Amazon Mechanical Turk, and may not reflect associations in the general public. Thus, these results alone should not be used to draw substantive conclusions.

Implicit association methods hold promise for improving our understanding of attitudes and practice behaviors of health professionals. Future validation work should consider further manipulation of the IAT stimuli, triangulation with empathy measures, professional behaviors, and patient outcomes, and larger and more generalizable samples both from the general community and within health professions at later stages of training and practice. If sufficient evidence for validity of the IAT scores is adduced, and particularly if IAT scores are associated with professional behaviors, the impact of providing IAT results to participants for reflection may also be studied as an educational intervention.

## Supporting information

S1 Dataset IAT scores from Amazon Mechanical Turk workers. (CSV)

S2 Dataset IAT scores from medical and nursing students. (CSV)

S1 Code IAT scoring code (R programming language). (R)
